# Breast-related effects of selective estrogen receptor modulators and tissue-selective estrogen complexes

**DOI:** 10.1186/bcr3677

**Published:** 2014-06-18

**Authors:** Carolyn L Smith, Richard J Santen, Barry Komm, Sebastian Mirkin

**Affiliations:** 1 Molecular and Cellular Biology, Baylor College of Medicine, One Baylor Plaza, Houston, TX 77030, USA; 2 University of Virginia School of Medicine, 450 Ray C. Hunt Drive, Fontaine Research Park, Charlottesville, VA 22908, USA; 3 Pfizer Inc, 500 Arcola Road, Collegeville, PA 19426, USA

## Abstract

A number of available treatments provide relief of menopausal symptoms and prevention of postmenopausal osteoporosis. However, as breast safety is a major concern, new options are needed, particularly agents with an improved mammary safety profile. Results from several large randomized and observational studies have shown an association between hormone therapy, particularly combined estrogen-progestin therapy, and a small increased risk of breast cancer and breast pain or tenderness. In addition, progestin-containing hormone therapy has been shown to increase mammographic breast density, which is an important risk factor for breast cancer. Selective estrogen receptor modulators (SERMs) provide bone protection, are generally well tolerated, and have demonstrated reductions in breast cancer risk, but do not relieve menopausal symptoms (that is, vasomotor symptoms). Tissue-selective estrogen complexes (TSECs) pair a SERM with one or more estrogens and aim to blend the positive effects of the components to provide relief of menopausal symptoms and prevention of postmenopausal osteoporosis without stimulating the breast or endometrium. One TSEC combination pairing conjugated estrogens (CEs) with the SERM bazedoxifene (BZA) has completed clinical development and is now available as an alternative option for menopausal therapy. Preclinical evidence suggests that CE/BZA induces inhibitory effects on breast tissue, and phase 3 clinical studies suggest breast neutrality, with no increases seen in breast tenderness, breast density, or cancer. In non-hysterectomized postmenopausal women, CE/BZA was associated with increased bone mineral density and relief of menopausal symptoms, along with endometrial safety. Taken together, these results support the potential of CE/BZA for the relief of menopausal symptoms and prevention of postmenopausal osteoporosis combined with breast and endometrial safety.

## Introduction

Vasomotor symptoms (VMSs) occur in up to 88% of women during the early years of menopause [1], and vulvar-vaginal atrophy (VVA) symptoms (for example, vaginal dryness, irritation, soreness, and dyspareunia) are reported by up to 50% of postmenopausal women [2]. Women also lose bone mineral density (BMD) after menopause, leading to increased risk of osteoporosis and fractures [3,4].

Hormone therapy (HT) comprises estrogen therapy (ET) for hysterectomized women and estrogen-progestin therapy (EPT) for women with a uterus. Although ET and EPT effectively treat VMS and VVA and prevent postmenopausal osteoporosis [5], some regimens are associated with breast safety and tolerability issues [6-8]. Other approved agents for prevention or treatment of postmenopausal osteoporosis - that is, raloxifene (RLX), bisphosphonates, calcitonin, parathyroid hormone, and denosumab - do not relieve menopausal symptoms.

Tissue-selective estrogen complexes (TSECs), which combine a selective estrogen receptor modulator (SERM) with one or more estrogens, represent a new therapeutic approach. This strategy aims to relieve menopausal symptoms and prevent postmenopausal osteoporosis without negatively impacting breast and endometrial safety. The recently approved TSEC pairing conjugated estrogens (CEs) with bazedoxifene (BZA) was evaluated in a series of phase 3 clinical trials in postmenopausal women [9-20]. A key issue is whether TSECs exert estrogenic or anti-estrogenic effects on the breast. This review summarizes the breast-related effects of HT, SERMs, and TSECs in postmenopausal women.

## Breast-related effects of estrogen therapy

In clinical settings, HT has been associated with breast safety issues, including a potential increase in breast cancer [6,8], increased mammographic breast density [21,22], and breast pain or tenderness [7]. Breast cancer risk appears to be greater with EPT than ET. The randomized, placebo-controlled Women’s Health Initiative (WHI) study initially reported a lower risk of invasive breast cancer after a mean of 6.8 years of ET (specifically CE) use (hazard ratio (HR) 0.77, 95% CI 0.57 to 1.06; *P* = 0.06), with separation of the Kaplan-Meier curves beginning at 2 years [23]. Despite early termination of the trial and discontinuation of ET by more than 90% of participants, reduction in breast cancer risk among those originally randomly assigned to ET persisted and became statistically significant during continued follow-up (HR 0.77, 95% CI 0.62 to 0.95; *P* = 0.02 at a median of 10.7 years of follow-up), and mortality among those with breast cancer was also reduced in the ET group (HR 0.62, 95% CI 0.39 to 0.97; *P* = 0.04) [24]. In contrast, the parallel, randomized, placebo-controlled WHI study of EPT found it to be associated with an increased risk of invasive breast cancer (HR 1.24, 95% CI 1.01 to 1.54; *P* = 0.003) at study termination (mean of 5.6 years) [8], which persisted in the postintervention period (HR 1.25, 95% CI 1.07 to 1.46; *P* = 0.004 at a mean of 11.0 years of follow-up) [25]. Furthermore, the WHI study found an increased risk of breast-cancer related mortality in the EPT group at long-term follow-up (HR 1.96, 95% CI 1.00 to 4.04; *P* = 0.049) [25].

The exact mechanism for an association between estrogens and breast cancer risk is not fully understood. One hypothesis argues that binding of estrogens to estrogen receptors (ERs) stimulates cellular proliferation, which increases the likelihood that DNA damage will be propagated during cell division, ultimately leading to breast carcinogenesis [26]. Another hypothesis is that estrogens are not oncogenic but promote existing occult tumors [27]. A third suggests that estrogen metabolites such as the catechol estrogens interact with DNA directly and have a carcinogenic effect in the breast [28].

Different types of estrogens may exhibit different estrogenic effects in breast tissue. For example, in a study comparing CE and estradiol on the growth and proliferation of MCF-7 breast cancer cells *in vitro*, estradiol was approximately 10 times more potent than CE for stimulating MCF-7 breast cancer overall cell growth and cellular proliferation [29]. In addition, estradiol inhibited apoptosis at a lower concentration than CE and, in gene expression studies, stimulated the expression of progesterone receptor (PR) and amphiregulin to a greater extent than CE [29]. In a separate study evaluating estradiol and CE on the growth of MCF-7 xenografts in a mouse model, estradiol - but not CE - stimulated tumor growth [30]. Estradiol also induced higher expression of a number of known ER target genes than did CE, which exhibited only weak estrogenic activity [30].

Despite progesterone’s anti-proliferative effects on the endometrium, it has been shown to have proliferative effects in the breast, independent of estrogen [31]. Preclinical data suggest a number of mechanisms by which progestins may increase breast cancer risk. One hypothesis is that progestogens activate the stem cell pool and accelerate tumor formation in the breast or that they convert some PR^+^ cells into basal ER^-^/PR^-^ stem cell-like cells [32]. The estrogen component of EPT may restore PR expression (which declines during postmenopause), allowing stimulation and amplification by the progestin component of these previously dormant stem cells [32]. In addition, progestins have been shown to stimulate production of vascular endothelial growth factor and therefore may promote tumor angiogenesis [33]. Recent studies have implicated progesterone in breast cancer cell proliferation through intracellular signaling via the PR and its various downstream targets and effectors [34,35]. There is also evidence that progestin-activated PR signaling leads to downregulation of the microRNA miR-16, a potent suppressor of breast cancer cell growth and proliferation [36]. Finally, there is evidence that progestins increase angiogenesis and decrease apoptosis via differential regulation of fibroblast growth factors, independent of PR signaling [37].

Increased mammographic breast density is a significant risk factor for breast cancer development [38,39], and high mammographic breast density may decrease sensitivity for detecting breast abnormalities [40]. New-onset breast tenderness occurs significantly more frequently in women receiving combined EPT compared with placebo or CE alone [41] and is associated with increased mammographic breast density [21] and subsequent breast cancer risk [41].

## Breast-related effects of selective estrogen receptor modulators

**Figure 1:**
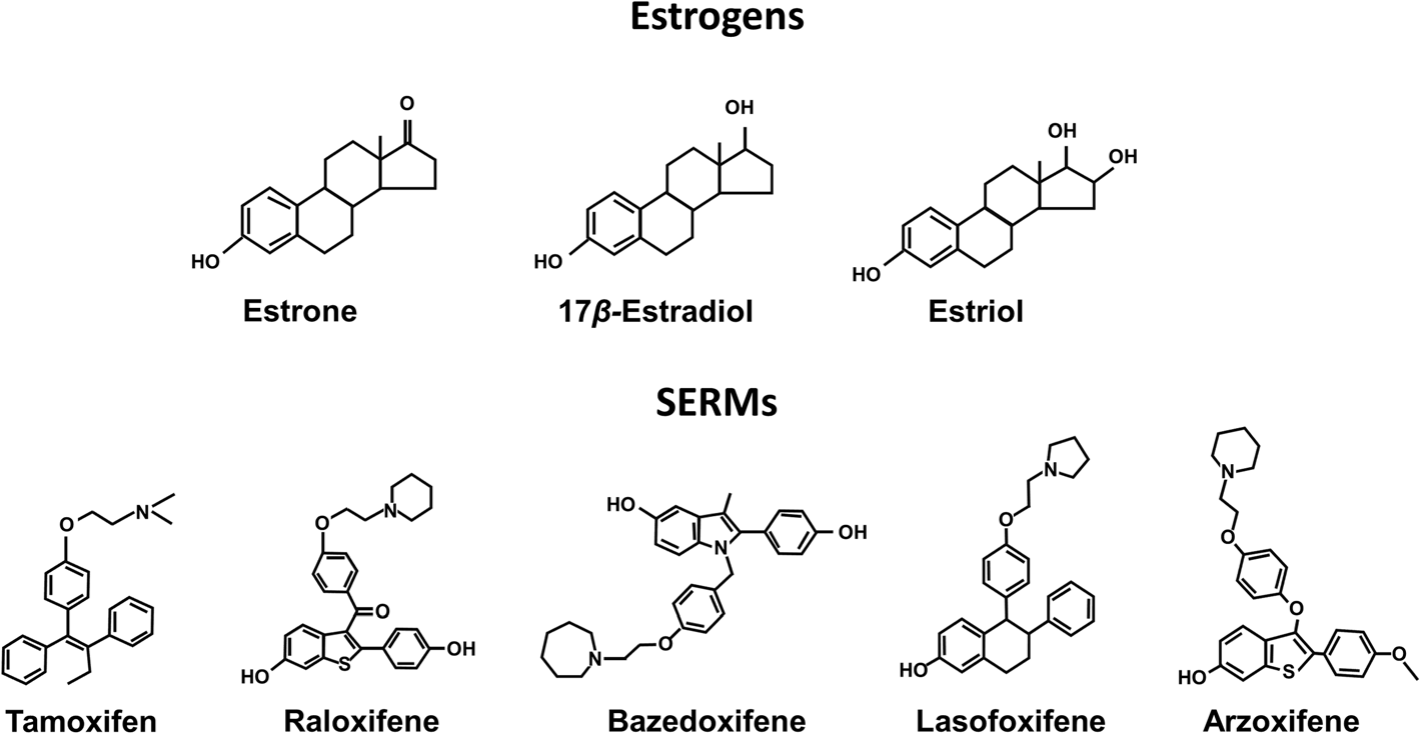
**Structural diversity of estrogens and selective estrogen receptor modulators (SERMs).** A chemically diverse group of SERMs and estrogens all function by binding to estrogen receptors.

**Figure 2:**
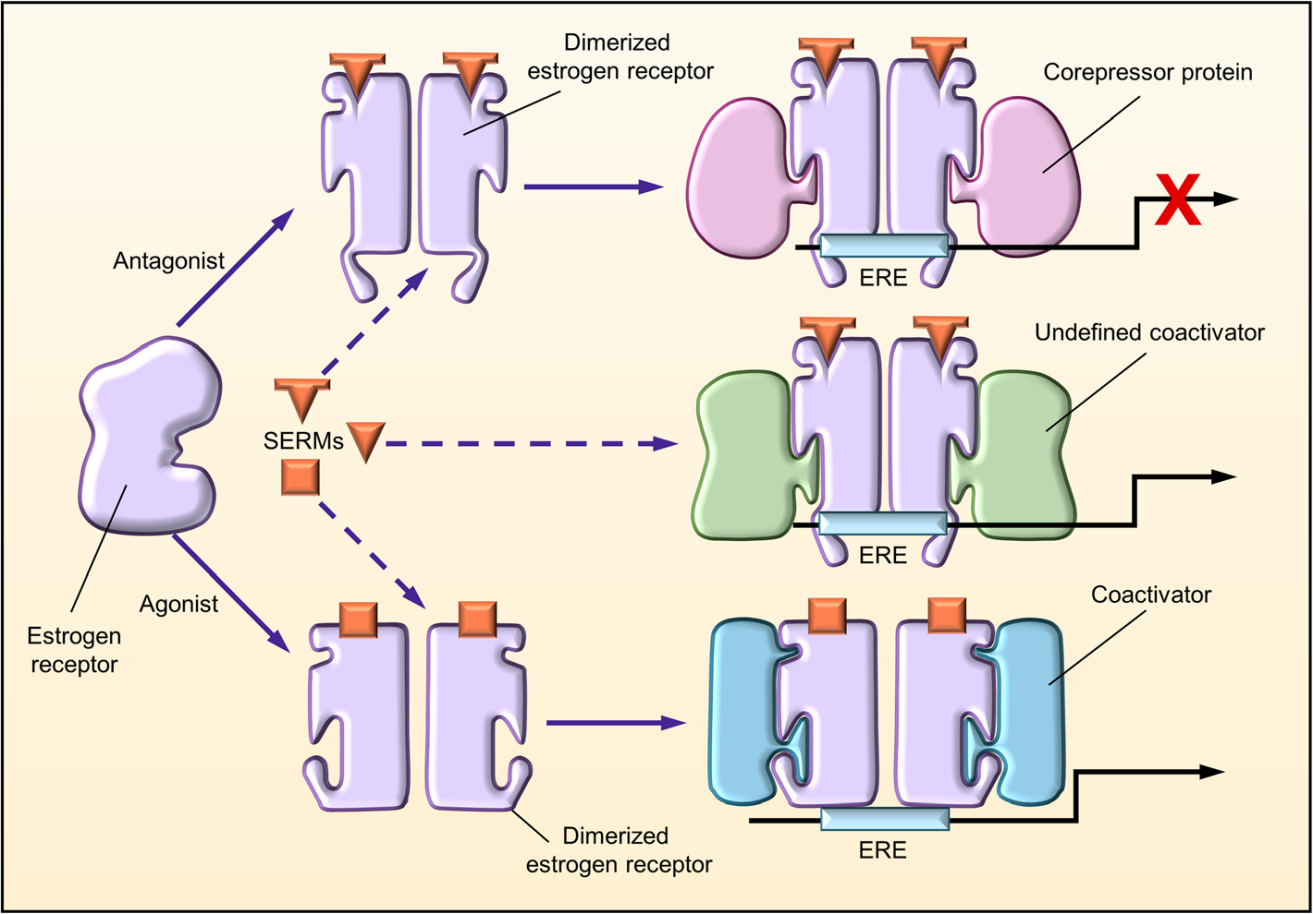
**Molecular activity of selective estrogen receptor modulators (SERMs) at estrogen receptors.** When a SERM binds to the estrogen receptor, the receptor adopts a unique conformation that allows dimerization and interaction with estrogen response elements (EREs) of the target genes. The unique conformational change induced by binding of the SERM may result in a distinct pattern of cofactor recruitment. Reprinted with permission from Elsevier [43].

SERMs are structurally diverse compounds that differ chemically from each other as well as from estrogens (Figure 1). Like estrogens, SERMs bind to the ER; however, whereas estrogens are ER agonists, SERMs exhibit selective agonist and antagonist effects depending on the target tissue [42]. After an individual SERM binds to the ER, the SERM-ER complex adopts a unique conformation (Figure 2) that may result in a distinctive pattern of cofactor recruitment [43]. Each SERM’s activity is derived from its unique ability to direct the structure of the receptor’s ligand-binding domain and consequently its interaction with coactivators or corepressors, resulting in cell- and tissue-specific alterations in gene expression [43]. Tamoxifen and RLX were initially classified as anti-estrogens that inhibited estrogen’s stimulatory actions in breast cancer cells [44]. However, early studies revealed that these agents could exhibit agonist or antagonist activities in different tissues, resulting in their re-classification as SERMs [44-46]. For example, although tamoxifen showed anti-estrogenic activity in the treatment and prevention of breast cancer, it also demonstrated estrogen agonist activity with its bone-protective and endometrial-stimulatory effects [47,48]. The mechanistic insights gained from studies of tamoxifen and RLX prompted the development of second- and third-generation SERMs, such as lasofoxifene (LAS), BZA, arzoxifene, and ospemifene [49]. Like tamoxifen, arzoxifene has been associated with positive bone effects and prevention of ER^+^ breast cancer but also stimulation of the endometrium [50-52], whereas ospemifene is indicated only for the treatment of moderate to severe dyspareunia, a symptom of VVA, due to menopause [53]. The impact of the other SERMs on the breast is discussed below.

### Tamoxifen

Tamoxifen, which has been in clinical use for approximately 40 years, is approved by the US Food and Drug Administration for treatment of metastatic breast cancer, adjuvant treatment of node-positive and axillary node-negative breast cancer, ductal carcinoma *in situ*, and breast cancer risk reduction in women at high risk [54]. Preclinical results demonstrated inhibitory effects of tamoxifen on growth of ER^+^ tumors [55,56], and an extensive body of clinical evidence supports the efficacy of tamoxifen in treatment and risk reduction of breast cancer [57]. Tamoxifen is associated with a reduction in breast density [58], which has been positively associated with reduced breast cancer risk [59]. For example, Cuzick and colleagues [59] showed that tamoxifen-treated patients with a reduction in breast density of at least 10% experienced a 63% reduction in breast cancer risk (odds ratio 0.37, 95% CI 0.20 to 0.69) but that tamoxifen-treated patients with a reduction in breast density of less than 10% showed no risk reduction. Although many of its effects are beneficial, tamoxifen has been associated with an increased risk of thromboembolic events and endometrial cancer, which has led to a search for alternative agents for treatment and prevention of breast cancer [57].

### Raloxifene

RLX is approved for prevention and treatment of postmenopausal osteoporosis and for reduction in risk of invasive breast cancer in postmenopausal women with osteoporosis or at high risk for invasive breast cancer [60]. In preclinical studies, RLX did not promote the proliferation of MCF-7 breast cells [61,62]. A study by Lewis-Wambi and colleagues [63] compared the effects of BZA, 4-hydroxytamoxifen, endoxifen, RLX, and fulvestrant (a pure anti-estrogen) on the growth of hormone-dependent and hormone-independent MCF-7 breast cancer cell lines. RLX inhibited estrogen-stimulated breast cancer cell proliferation but did not inhibit proliferation of hormone-independent breast cancer cells. RLX is generally an ER antagonist in breast tissue; however, RLX may stimulate expression of certain ER genes. For example, in ovariectomized (OVX) mice, RLX upregulated expression of the mammary gland expression marker indoleamine-pyrrole 2,3 dioxygenase compared with vehicle control, which is indicative of ER agonist activity [64]. Nonetheless, clinical studies consistently show a reduced incidence of breast cancer in women receiving RLX [65-68]. For example, in the randomized, double-blind, Multiple Outcomes of Raloxifene Evaluation (MORE) trial of postmenopausal women with osteoporosis (n = 7,705), RLX decreased the risk of invasive breast cancer by 72% over 4 years compared with placebo (PBO) (relative risk (RR) 0.28, 95% CI 0.17 to 0.46) [65]. In the 4-year Continuing Outcomes Relevant to Evista (CORE) extension of the MORE study (n = 4,011), reduced risk of invasive breast cancer by RLX was maintained versus PBO (HR 0.41, 95% CI 0.24 to 0.71) [66]. At long-term follow-up (n = 19,490) of the randomized, double-blind Study of Tamoxifen and Raloxifene trial, RLX was less effective than tamoxifen for prevention of invasive breast cancer (RR 1.24, 95% CI 1.05 to 1.47) and ductal carcinoma *in situ* (RR 1.22, 95% CI 0.88 to 1.69) [68]. The incidence of breast pain was similar with RLX and PBO in the MORE [65] and CORE [66] studies, but in the Euralox-1 study of healthy postmenopausal women (n = 1,008), the incidence of breast pain was significantly lower with RLX than with HT (1.8% versus 26.5%, respectively; *P* <0.001) [69]. A recent review concluded that RLX did not increase or decrease mammographic breast density [70]; however, many of the studies assessing the effect of RLX on breast density have been relatively small (fewer than 200 patients), and methods of measuring breast density have not been consistent [71-73].

### Lasofoxifene

LAS was developed for treatment of vaginal atrophy and prevention and treatment of postmenopausal osteoporosis [74]. LAS exhibited anti-proliferative effects in estrogen-sensitive MCF-7 breast cancer cells [62,75] and did not change the histology of mammary tissue in OVX cynomolgus macaques [76]. The 5-year Postmenopausal Evaluation and Risk-reduction with Lasofoxifene study (n = 8,556) demonstrated an 81% reduction in risk of total ER^+^ breast cancer and an 83% reduction in risk of invasive ER^+^ breast cancer with LAS 0.5 mg/day versus PBO (*P* <0.001) [77]. Pooled results from two identical phase 3 studies of varying doses of LAS in postmenopausal women (n = 1,907) showed no increase in breast density or breast pain with LAS versus PBO [78]. LAS received European Union (EU) marketing authorization from the European Commission in 2009. A series of industry acquisitions, asset shifts, and licensing of product rights has since ensued, delaying its launch. Since LAS did not become commercially available within 3 years, its EU approval has lapsed but may be reapplied for eventually or it may become available as an unlicensed medicinal product available by physician request for patients who are not candidates for approved therapies [79,80].

## Bazedoxifene

BZA is a newer-generation SERM that has demonstrated efficacy for the prevention and treatment of postmenopausal osteoporosis [81,82]. Its development was prompted by the need for improved SERMs that could protect the skeleton, improve lipid profile, reduce hot flush frequency and severity, reduce vaginal dryness and atrophy, and maintain bladder function without stimulating the endometrium or breast [83]. During preclinical development, BZA was shown to be a functionally active ER ligand with beneficial effects on bone and total cholesterol, antagonist activity in the breast, and neutral effects on the endometrium; however, it did not inhibit vasomotor response when given at a bone-protective dose [83]. Although a discussion of the full range of effects of BZA and other SERMs is beyond the scope of this article, a review of this topic was recently published [84], as was a review of the differential effects of menopausal therapies on the endometrium [85].

Further preclinical investigations confirmed that BZA acts as an estrogen antagonist in the breast. In MCF-7 proliferation assays, BZA did not stimulate cell proliferation and potently inhibited estradiol-stimulated proliferation [61]. In the previously described study by Lewis-Wambi and colleagues [63], all of the compounds studied inhibited estradiol-stimulated breast cancer cell proliferation, but only BZA and fulvestrant significantly inhibited the growth of hormone-independent MCF-7:5C cells [63]. Growth inhibition of MCF-7:5C with BZA was associated with cell cycle arrest and downregulation of cyclin D1 and ERα [63]. In a separate study evaluating the effects of BZA on the growth of breast cancer xenografts in mouse models, BZA inhibited the growth of tamoxifen-sensitive and tamoxifen-resistant tumor xenografts [86]. Unlike other SERMs, BZA downregulates ER expression and therefore shares some properties of selective estrogen receptor degraders, such as the pure anti-estrogen fulvestrant [86]. The extent to which this contributes to growth inhibition of tumor xenografts is unclear, but BZA exhibits antagonist activity in breast cancer cells *in vitro* independent of ER degradation [86].

BZA’s lack of ER-stimulatory activity in the breast is reinforced by data from phase 3, randomized, double-blind studies in postmenopausal women [87-89]; however, these studies were not sufficiently powered to detect breast cancer prevention. In a 2-year trial of varying doses of BZA, RLX 60 mg, and PBO in healthy postmenopausal women at risk for osteoporosis (n = 1,583), rates of breast carcinoma (0% to 0.6%) and breast pain (2.6% to 3.7%) were low and similar among treatment groups [87]. A phase 3, randomized, double-blind, 3-year trial of BZA (20 or 40 mg), RLX 60 mg, or PBO in women with osteoporosis (n = 7,492) showed no significant difference in the incidence of breast carcinoma and breast cysts with BZA compared with PBO or RLX; however, there was a significantly lower incidence of fibrocystic breast disease with BZA 20 or 40 mg (0.3% and 0.2%, respectively) versus RLX (0.8%; both *P* ≤0.05) [88,89]. Breast pain incidence was similar among groups, with rates ranging from 2.4% to 3.0% [88,89]. In a retrospective ancillary analysis of a subset of women (n = 444) from this study, mean percentage changes in breast density from baseline were low and similar among groups [90]. Consistent with the 3-year results, BZA showed a neutral effect on the breast at 5 years [91] and 7 years [92] of therapy.

## Breast-related effects of the tissue-selective estrogen complex

As described above, SERMs are generally well tolerated and have not been shown to stimulate the breast. However, no SERM alone achieves an optimal balance of providing ER agonist activity to relieve menopausal symptoms and prevent bone loss while exerting antagonist effects on the breast. By pairing a SERM with one or more estrogens, the TSEC blends estrogenic properties with the tissue-selective activity of a SERM [93] with the goals of relieving menopausal symptoms and preventing bone loss while minimizing estrogenic effects on the endometrium and breast [93]. In comparison with EPT, a more favorable breast safety profile was expected for TSECs given the lack of a progestogen component, which (as noted above) has been associated with an increased risk of breast cancer compared with estrogens alone [94].

Molecular activity of the TSEC components at the receptor level may help explain differences in their activity compared with that of individual estrogens and SERMs. With a TSEC, competition of the SERM and estrogen components for binding to ERs is expected to result in unique combinations of ERs with conformations reflecting both SERM and estrogens. Furthermore, since SERMs and estrogens both bind to ERs, it is possible that their combination facilitates formation of novel receptor dimers that bind to both SERMs and estrogens rather than one or the other. Results of a recent study support the cooperative interaction of SERMs and estrogens through a heterodimeric complex [95]. Using an *in vitro* reporter gene assay, the cooperative control of gene expression by ER agonists (for example, CE and 17β-estradiol) and antagonists (for example, tamoxifen, RLX, BZA, and fulvestrant) was shown to be mediated through an ER heteroligand dimer complex, in which the ER agonist binds to one dimer subunit and the antagonist binds to the other [95]. The actions of TSEC within a given cell type may also reflect the net effect of TSECs on both receptor and cell environment integrated at the level of gene expression (for example, estrogens may modify the activity or expression of a cofactor that could promote SERM activity and *vice versa*) [96].

### Preclinical comparisons of different tissue-selective estrogen complexes

The breast-related effects of different TSECs, combining RLX, LAS, or BZA with one or more estrogens, have been evaluated in preclinical studies (Table 1) [30,62,64,97-99].

**Table 1:** Preclinical results

**Study reference**	**Study design/model**	**Key results**
*In vitro* studies		
Berrodin *et al*. [97] (2009)	Multiplex ERα-cofactor peptide interaction assay	LAS and RLX completely inhibited CE-mediated recruitment of all cofactor peptides to the ERα-ligand-binding domain, whereas CE/BZA inhibited the CE-mediated recruitment of some, but not all, peptides.
	GeneChip microarray	
		CE/BZA gene expression profile was significantly different from CE/LAS or CE/RLX.
		CE/RLX, CE/LAS, and CE/BZA antagonized genes involved in cell cycle regulation and growth hormone signaling; CE/RLX and CE/LAS also antagonized expression of a set of CE-regulated genes not affected by CE/BZA.
Chang *et al*. [62] (2010)	MCF-7 cell proliferation assays	CE/RLX, CE/LAS, and CE/BZA all antagonized CE-stimulated proliferation, with antagonism levels in the following order: BZA > RLX > LAS.
	GeneChip microarray	
		CE/BZA gene expression profile was significantly different from CE/LAS or CE/RLX.
*In vivo* studies		
Crabtree *et al*. [64] (2008)	Ovariectomized female mice	BZA and RLX (not LAS) reduced estradiol-induced mammary gland end bud proliferation.
		Estrogen-responsive marker studies in the mammary gland showed that BZA, RLX, and LAS all function as ER antagonists but have different degrees of agonist activity.
Peano *et al*. [98] (2009)	Ovariectomized mice	BZA completely inhibited CE-induced increases in ductal tree branch points; RLX and LAS only partially inhibited CE-induced effects.
Song *et al*. [30] (2012)	Ovariectomized mice with human MCF-7 breast cancer xenografts	BZA blocked the estrogenic effects of CE and estradiol (including ductal length, terminal end bud development, proliferation, apoptosis, and gene expression changes).
		BZA inhibited estradiol-induced tumor growth and weight.
Ethun *et al*. [99] (2012)	Ovariectomized cynomolgus monkeys	CE/BZA antagonized CE-stimulatory effects on total breast epithelial density, Ki67 staining, markers of ERα activity, and lobular size.
		BZA alone had neutral effects on all outcomes.

BZA, bazedoxifene; CE, conjugated estrogens; ER, estrogen receptor; LAS, lasofoxifene; RLX, raloxifene.

#### *In vitro* studies

Berrodin and colleagues [97] evaluated the effects of BZA, RLX, and LAS on CE-mediated recruitment of 43 cofactor peptides to the ERα-ligand-binding domain by a multiplex biochemical assay. LAS and RLX in combination with CE completely inhibited the CE-mediated recruitment of all peptides [97]. In contrast, BZA in combination with CE inhibited the CE-mediated recruitment of some, but not all, of the evaluated cofactor peptides, indicating the potential for tissue selectivity with CE/BZA and suggesting that CE/BZA induces different conformations of ERα than either CE/RLX or CE/LAS [97]. The investigators also studied gene expression profiles in MCF-7 breast cancer cells and found that CE/BZA exhibited an expression profile for a subset of genes in the global gene expression profile that maintained some of the characteristics of CE alone, whereas CE/RLX and CE/LAS completely antagonized expression of this subset of CE-regulated genes [97]. The CE-regulated genes antagonized by all three of these SERMs were involved in cell cycle regulation and cell-to-cell signaling [62,97]. Results from microarray studies comparing gene expression profiles of BZA, RLX, and LAS alone and in combination with CE in MCF-7 human breast cancer cells also supported a broad range of differences in gene expression patterns across the different SERM and TSEC combinations [62,96]. In an MCF-7 cell proliferation study, RLX, LAS, and BZA all significantly antagonized CE-stimulated proliferation of breast cancer cells; BZA exhibited similar or better efficacy at inhibiting MCF-7 cell growth than the other SERMs evaluated [62].

#### *In vivo* studies

Results of *in vivo* preclinical studies provide further evidence of differences in the activity of TSECs combining different SERMs with CE (Table 1) [64,98]. In the mammary gland of OVX mice, treatment with BZA and RLX (but not LAS), when given in combination with estradiol, reduced estradiol-induced mammary gland end bud proliferation [64]. In a separate study of the effects of BZA, RLX, and LAS alone and in combination with CE on mammary gland morphology in OVX sexually immature mice, BZA was the only SERM that completely inhibited CE-induced effects [98]. CE activity in the breast and uterus was antagonized by BZA to a greater extent than by RLX or LAS [98].

Overall, preclinical data support the concept that TSECs exhibit distinct pharmacologic profiles. In many tissues, BZA and CE exhibit opposite effects on shared ER target genes (for example, growth-related genes may be upregulated by estrogens and downregulated by BZA), whereas some genes (for example, *FOS*, *CYR61*, and *ZNF10*) are regulated in the same direction by both BZA and CE [62]. These observations suggest that BZA selectively antagonizes certain aspects of CE action but may work in concert with CE at a subset of genes [62]. In contrast, results of gene expression profiling studies indicate that RLX or LAS in combination with CE antagonizes the expression of a greater number of CE-regulated genes, which theoretically could limit CE’s beneficial effects (for example, on vasomotor symptoms) [97]. Further preclinical and clinical data for CE/BZA, the only TSEC to complete clinical development, will be discussed below.

### Conjugated estrogens/bazedoxifene

#### Additional preclinical data

The breast safety profile of CE/BZA has been evaluated in murine and primate models [30,99]. In a study comparing estradiol, CE, and BZA, BZA blocked the estrogenic effects of CE or estradiol on ductal length, terminal end bud development, proliferation, apoptosis, and gene expression changes on mammary gland tissue and inhibited the growth and weight increase of tumors in human MCF-7 xenografts in OVX mice [30]. In the mammary gland of OVX cynomolgus macaques, CE stimulation of total breast epithelial density, Ki67 staining, markers of ERα activity, and lobular size were antagonized by treatment with CE/BZA [99]. On the basis of gene markers of cell proliferation or cell cycle progression, both BZA and CE/BZA lacked estrogen activity in the breast. The investigators suggested that ERα protein degradation may play a role in the inhibitory breast effects of BZA [99]. Along with the previously described results of comparative studies of CE/BZA and other TSEC combinations, these results suggest that CE/BZA can antagonize estrogenic activity in normal and cancerous breast tissue.

### Clinical studies

The efficacy and safety of CE/BZA were evaluated in the Selective estrogens, Menopause, And Response to Therapy (SMART) trials, five randomized, double-blind, placebo- and active-controlled, phase 3 trials that enrolled postmenopausal women with a uterus (Table 2) [9-19,100-102]. Overall, the SMART trials demonstrated increased BMD and relief of VMS and VVA with CE/BZA treatment (Table 2) while ensuring endometrial safety and breast protection (Table 3). On the basis of these studies, CE 0.45 mg/BZA 20 mg once daily was approved in 2013 by the US Food and Drug Administration for treatment of moderate to severe VMSs associated with menopause and for prevention of postmenopausal osteoporosis in women with a uterus [103].

**Table 2:** The SMART clinical trial program

**Study**	**Study design**	**Main inclusion criteria**	**Number of patients randomly assigned**	**Treatment groups**	**Primary endpoint**	**Key results**
SMART-1 [9-12,100]	2-year, randomized, double-blind, multicenter, placebo- and active (RLX)-controlled, phase 3 trial	Age 40–75 years Postmenopausal (≥12 months amenorrhea, FSH ≥30 mIU/mL, and 17β-E2 ≤183.5 pmol/L)	3,544 OSS I: 1,454 OSS II: 861 [12]	CE 0.625 mg/BZA 10 mg	Incidence of endometrial hyperplasia at 1 year	CE 0.45 and 0.625 mg/BZA 20 and 40 mg showed low rates (<1%) of endometrial hyperplasia [9]
		With a uterus				
		No evidence of endometrial hyperplasia		CE 0.625 mg/BZA 20 mg		
		BMI ≤32.2 kg/m^2^				
		OSS I: >5 YSM with a baseline BMD T-score between -1 and -2.5 and ≥1 additional risk factor for osteoporosis [12]				
				CE 0.625 mg/BZA 40 mg		Other outcomes Incidence of abnormal mammograms at 2 years: 4.4% with CE 0.45 mg/BZA 20 mg, 4.2% with CE 0.625 mg/BZA 20 mg, 3.4% with RLX, and 2.6% with PBO [100]
		OSS II: 1–5 YSM with ≥1 risk factor for osteoporosis [12]				
						CE 0.625 and 0.45 mg/BZA 20 and 40 mg associated with rates of cumulative amenorrhea similar to PBO (>83% (cycles 1–13) and >93% (cycles 10–13)); bleeding and spotting rates similar to PBO [11]
				CE 0.45 mg/BZA 10 mg		
				CE 0.45 mg/BZA 20 mg		
				CE 0.45 mg/BZA 40 mg		
				RLX 60 mg PBO		
						CE 0.45 and 0.625 mg/BZA 20 mg significantly reduced number (*P* <0.05 for both) and severity (*P* <0.001 for both) of hot flushes vs. PBO at week 12 [10]
						CE 0.625 and 0.45 mg/BZA 20 mg significantly reduced VVA vs. PBO at month 24, and CE 0.625 mg/BZA 20 mg significantly reduced the incidence of dyspareunia at weeks 5–12 [10]
						CE 0.45 and 0.625 mg/BZA 20 mg significantly improved LDL and HDL cholesterol vs. PBO (*P* <0.01 for all) at month 24 [10]
						OSSs
						In both OSSs, CE/BZA was associated with significant BMD increases at lumbar spine (*P* <0.001) and total hip (*P* <0.01), with significant decreases in bone turnover markers [12]
SMART-2 [13,14]	12-week, multicenter, double-blind, randomized, placebo-controlled, phase 3 trial	Age 40–65 years	332	CE 0.45 mg/BZA 20 mg	Change from baseline in average daily number of moderate and severe hot flushes and the severity of hot flushes at weeks 4 and 12	CE/BZA at both doses significantly reduced the number and severity of hot flushes vs. PBO at weeks 4 and 12 (*P* <0.001 for all) [13]
		Postmenopausal (≥12 months amenorrhea or 6 months amenorrhea with FSH >40 mIU/mL)		CE 0.625 mg/BZA 20 mg PBO		
						Other outcomes
		With a uterus				
		BMI ≤34.0 kg/m^2^				Women treated with CE/BZA experienced significant improvements in sleep parameters and overall menopause-related and vasomotor HR-QOL [14]
		≥7 moderate to severe hot flushes per day or ≥50 per week				
SMART-3 [15,16]	12-week, multicenter, double-blind, randomized, placebo- and active	Age 40–65 years	664	CE 0.45 mg/BZA 20 mg	4 co-primary endpoints: change from baseline in (1) proportion of vaginal superficial cells,	CE/BZA at both doses significantly (*P* <0.01) increased superficial cells and decreased parabasal cells vs. PBO [15]
		Postmenopausal (≥12 months amenorrhea or 6 months amenorrhea with FSH >40 mIU/mL)		CE 0.625 mg/BZA 20 mg		
	(BZA)-controlled, phase 3 trial					
				BZA 20 mg		
		With a uterus		PBO	(2) proportion of parabasal cells, (3) vaginal pH, and (4) severity of the most bothersome vulvar-vaginal symptom at week 12	CE 0.625 mg/BZA 20 mg was associated with a significant decrease in vaginal pH from baseline (*P* <0.001), a decrease that was significantly greater than that seen with placebo (*P* <0.001) [15]
		BMI ≤34.0 kg/m^2^				
		≤5% or less superficial cells on vaginal cytological smear				
						CE 0.625 mg/BZA 20 mg was associated with improvement in most bothersome vulvar-vaginal symptom at week 12 vs. PBO (*P* <0.05) [15]
		Vaginal pH >5				
		≥1 moderate to severe bothersome vulvar-vaginal symptom				
						Other outcomes
		No endometrial hyperplasia, estrogen-dependent neoplasia, undiagnosed vaginal bleeding, or focal endometrial abnormality on transvaginal ultrasound				CE 0.45 and 0.625 mg/BZA 20 mg significantly improved sexual function measured by ASEX (*P* <0.05) vs. PBO [16]
						CE 0.45 and 0.625 mg/BZA 20 mg significantly improved vasomotor function, sexual function, and overall menopause-related HR-QOL measured by MENQOL (*P* <0.01) vs. PBO [16]
SMART-4 [17]	1-year, multicenter, double-blind, randomized, placebo- and active- (CE/MPA) controlled, phase 3 study	Age 40–59 years	1,083	CE 0.45 mg/BZA 20 mg	Incidence of endometrial hyperplasia at 1 year	There were 3 cases of endometrial hyperplasia in the CE 0.625 mg/BZA 20 mg group and none in the other groups
					OSS: mean percentage change from baseline in lumbar spine BMD at 1 year	
						All active treatments produced significant increases from baseline in lumbar spine and total hip BMD compared with PBO (*P* <0.001)
		Postmenopausal (≥12 months amenorrhea or 6 months amenorrhea with FSH >40 mIU/mL)		CE 0.625 mg/BZA 20 mg		
				CE 0.45 mg/MPA 1.5 mg PBO		
		With a uterus				
		BMI ≤34.0 kg/m^2^				
		No history of endometrial hyperplasia or undiagnosed vaginal bleeding				
		OSS: ≤5 years amenorrhea 2 evaluable BMD scans of lumbar spine differing by <5% and hip differing by <7.5%				
		No osteoporosis or fragility fractures				
SMART-5 [18,19,101,102]	1-year, multicenter, double-blind, randomized, placebo-, and active- (CE/MPA) controlled, phase 3 trial	Aged 40–65 years	1,843	CE 0.45 mg/BZA 20 mg	Incidence of endometrial hyperplasia and mean percentage change in lumbar spine BMD at 12 months	Incidence of endometrial hyperplasia with CE 0.45 and 0.625 mg/BZA 20 mg was low (≤0.3%) and similar to that with PBO and CE 0.45 mg/MPA 1.5 mg [102]
		Postmenopausal (≥12 months amenorrhea or 6 months amenorrhea with FSH >40 mIU/mL)				
				CE 0.625 mg/BZA 20 mg		
		With a uterus				
		BMI ≤34.0 kg/m^2^		BZA 20 mg		
		Acceptable endometrial biopsy		CE 0.45 mg/MPA 1.5 mg PBO		
		Seeking treatment for menopausal symptoms				
		Sleep/HR-QOL substudy: bothered by hot flushes/night sweats plus sleep interruptions				
						CE 0.45 and 0.625 mg/BZA 20 mg were associated with significant improvements in lumbar spine BMD vs. PBO at 1 year (*P* <0.001) [101]
						Other outcomes
						CE 0.45 and 0.625 mg/BZA 20 mg were non-inferior to PBO in percentage change in mammographic breast density [18]
						In the sleep/HR-QOL substudy, both doses of CE/BZA were associated with significant improvements in sleep parameters and HRQOL at 1 year [19]

17β-E2, 17β-estradiol; ASEX, Arizona Sexual Experiences Scale; BMD, bone mineral density; BMI, body mass index; BZA, bazedoxifene; CE, conjugated estrogens; FSH, follicle-stimulating hormone; HDL, high-density lipoprotein; HR-QOL, health-related quality of life; LDL, low-density lipoprotein; MENQOL, Menopause-specific Quality of Life; MPA, medroxyprogesterone acetate; OSS, Osteoporosis Substudy; PBO, placebo; RLX, raloxifene; SMART, Selective estrogens, Menopause, And Response to Therapy; VVA, vulvar-vaginal atrophy; YSM, years since menopause.

**Table 3:** Breast safety results from the SMART clinical trial program

	**SMART-1**	**SMART-2**	**SMART-3**	**SMART-4**	**SMART-5**
Breast pain/tenderness	No significant differences in incidence of breast pain for any dose of CE/BZA, RLX 60 mg, or PBO [10]	No significant difference in number of women reporting ≥1 day of breast pain between CE/BZA and PBO [13]	Incidence of breast pain with CE 0.45 mg/BZA 20 mg and 0.625 mg/BZA 20 mg not significantly different from PBO [15]	No significant difference in number of women reporting ≥1 day of breast pain between CE/BZA and PBO	Rates of breast tenderness with CE 0.45 mg/BZA 20 mg and 0.625 mg/BZA 20 mg from 5.8%-9.4%, similar to PBO (5.4%-8.6%) [18]
					Rates of breast tenderness with CE 0.45 mg/BZA 20 mg and 0.625 mg/BZA 20 mg were significantly lower than with CE 0.45 mg/MPA 1.5 mg (7.3%-24.3%; *P* <0.001) [18]
				Compared with CE 0.45 mg/MPA 1.5 mg, breast pain incidence was significantly lower with CE 0.45 mg/BZA 20 mg at weeks 5–8 and 9–12 (*P* <0.05) and for CE 0.625 mg/BZA 20 mg at weeks 1–4, 4–8, and 9–12 (*P* <0.01) [17]	
Abnormal mammogram findings	n (%) at 2 years: CE 0.45 mg/BZA 20 mg, 13 (4.4%)	ND	ND	ND	n (%) at 1 year: CE 0.45 mg/BZA 20 mg, 4 (0.9%)
	CE 0.625 mg/BZA 20 mg, 11 (4.2%)				CE 0.625 mg/BZA 20 mg, 2 (0.4%)
	RLX 60 mg, 9 (3.4%)				BZA 20 mg, 1 (0.4%)
	PBO, 7 (2.6%) [100]				CE 0.45 mg/MPA 1.5 mg, 3 (1.4%)
					PBO, 1 (0.2%) [18]
Breast density changes	No significant differences between groups in breast density	ND	ND	ND	No significant differences from PBO with CE 0.45 mg/BZA 20 mg or 0.625 mg/BZA 20 mg; CE 0.45 mg/MPA 1.5 mg significantly increased breast density vs. PBO (*P* <0.001)
	Mean (SD) percentage change from baseline in percentage breast density at 2 years: CE 0.45 mg/BZA 20 mg, -0.39% (1.75%)				
					Adjusted percentage change from baseline in percentage breast density at 1 year, mean (SD): CE 0.45 mg/BZA 20 mg, -0.38% (0.22%)
	CE 0.625 mg/BZA 20 mg, -0.05% (1.68%)				
	RLX 60 mg, -0.23% (1.76%)				
	PBO, -0.42% (1.72%) [100]				CE 0.625 mg/BZA 20 mg, -0.44% (0.22%)
					BZA 20 mg, -0.24% (0.30%)
					CE 0.45 mg/MPA 1.5 mg, 1.60% (0.35%)
					PBO, -0.32% (0.23%) [18]

BZA, bazedoxifene; CE, conjugated estrogens; MPA, medroxyprogesterone acetate; ND, not determined; PBO, placebo; RLX, raloxifene; SD, standard deviation; SMART, Selective estrogens, Menopause, And Response to Therapy.

#### Breast safety outcomes from clinical studies

The incidence of breast-related adverse events was low and similar for CE 0.45 mg/BZA 20 mg compared with PBO in a pooled analysis of the SMART-1 to -3 trials [104]. In SMART-1 (n = 3,397), the incidences of abnormal mammograms at 2 years were similar for CE 0.45 mg/BZA 20 mg (4.4%) and PBO (2.6%) [100]. Similarly, in SMART-5 (a placebo-controlled comparison of CE/BZA and HT, n = 1,843), incidences of abnormal mammograms at 1 year were similar for CE 0.45 mg/BZA 20 mg (0.9%) and PBO (0.2%) [18].

Breast cancer rates were low across the SMART studies [18,104], although it is important to note that these studies were not powered to demonstrate breast cancer prevention. In a pooled analysis of the SMART-1 to -3 studies, the rate of breast cancer in the CE 0.45 mg/BZA 20 mg group (0.25%) was similar to that in the placebo group (0.17%) [104].

CE/BZA does not appear to affect breast density. In an ancillary retrospective study of SMART-1 (n = 507), mean mammographic breast density changes at 2 years with CE 0.45 mg/BZA 20 mg (-0.39%) were similar to those with PBO (-0.42%) and RLX 60 mg (-0.23%) [100]. Similarly, in the breast density substudy of SMART-5, CE 0.45 mg/BZA 20 mg was not associated with increased breast density and demonstrated non-inferiority compared with PBO at 12 months [18]. In contrast, CE 0.45 mg/medroxyprogesterone acetate (MPA) 1.5 mg was associated with a significant increase in breast density at 12 months versus PBO [18].

#### Breast pain and tenderness outcomes from clinical studies

There was no significant difference between the CE 0.45 mg/BZA 20 mg and PBO groups in the percentage of women reporting breast pain or tenderness in any SMART trial [10,13,15,17,18]. In SMART-1 (n = 3,397), the incidence of breast pain among the CE/BZA, RLX, and PBO group was not significantly different [10]. In the SMART-2 (n = 332), SMART-3 (n = 664), and SMART-4 (n = 1,061) trials, there were no significant differences in the number of women reporting at least 1 day of breast pain between the CE/BZA and PBO groups [13,15,17]. In SMART-5 (n = 1,843), the incidence of breast tenderness with CE/BZA was similar to PBO and significantly lower than with CE/MPA (*P* <0.001) [18]. Thus, the clinical data support the favorable breast-related safety and tolerability profile of CE/BZA in postmenopausal women with a uterus.

## Conclusions

A number of available treatments provide effective relief of VMS and VVA along with prevention of postmenopausal osteoporosis, but new options with improved breast-related safety and tolerability are needed. Results from large randomized and observational studies have shown an association between HT, particularly EPT, and increased risk of breast cancer [6-8]. HT use by postmenopausal women has been associated with increased mammographic breast density [21,22], which is a risk factor for breast cancer [38,39]. Breast pain and tenderness are also a concern with HT.

SERMs are generally well tolerated and have demonstrated positive effects on the breast but do not relieve menopausal symptoms. A TSEC aims to take advantage of the positive effects of both the SERM and the estrogen components to treat menopausal symptoms and prevent postmenopausal osteoporosis without stimulating the breast or endometrium. Strong preclinical evidence supports the breast safety of CE/BZA. Clinically, CE/BZA has shown no or minimal increased risk of breast effects, and the results of phase 3 clinical trials have demonstrated a favorable breast-related safety and tolerability profile in non-hysterectomized postmenopausal women. Additional studies are necessary to elucidate the effects, if any, of HT, SERMs, and TSECs on occult pre-existing breast tumors.

## Abbreviations

BMD: Bone mineral density; BZA: Bazedoxifene; CE: Conjugated estrogen; CORE: Continuing Outcomes Relevant to Evista; EPT: Estrogen-progestin therapy; ER: Estrogen receptor; ET: Estrogen therapy; EU: European Union; HR: Hazard ratio; HT: Hormone therapy; LAS: Lasofoxifene; MORE: Multiple outcomes of raloxifene evaluation; MPA: Medroxyprogesterone acetate; OVX: Ovariectomized; PBO: Placebo; R: Progesterone receptor; RLX: Raloxifene; RR: Relative risk; SERM: Selective estrogen receptor modulator; SMART: Selective estrogens, menopause, and response to therapy; TSEC: Tissue-selective estrogen complex; VMS: Vasomotor symptom; VVA: Vulvar-vaginal atrophy; WHI: Women’s Health Initiative.

## Competing interests

BK is a full-time employee of Pfizer. SM was a full-time employee of Pfizer at the time of manuscript development. RJS serves on an advisory board for Pfizer; is a consultant for Pfizer, Teva (Petah Tikva, Central District, Israel), and Novo Nordisk (Bagsvaerd, Denmark); and has received a grant from Pfizer to study the *in vitro* and *in vivo* effects of TSECs. CLS serves on an advisory board for Pfizer and has received research funding from Pfizer.

## Authors’ contributions

All authors were involved in drafting the manuscript or critically revising it for important intellectual content and have read and approved the final manuscript.
